# Preface

**DOI:** 10.2174/1570159X2201230921145316

**Published:** 2023-09-21

**Authors:** Ferdinando Nicoletti

**Affiliations:** 1 Full Pofessor of Pharmacology, University Sapienza of Rome, IRCCS Neuromed, Pozzilli, Italy

It has been a great privilege to act as EIC of *Current Neuropharmacology* and collaborate with the team of Bentham Science Publishers in 2023. I wish to express my gratitude to Miss Rabia Moin and all other members of the Bentham team. As always, Rabia has been fantastic in coordinating the editorial activity and addressing the authors' requests, doing her best to accelerate the publication of accepted manuscripts. Rabia is a combination of efficiency, professionalism, and kindness, and she is the ideal linking bridge between Authors and the Editorial Board of the journal. I am also grateful to all members of the Editorial Board, who worked hard for the journal's success. For the coming year, we have decided to accept only a limited number of top-level research articles or clinical studies, subjected to evaluation by a minimum of four reviewers who are esteemed leaders in the field. Review articles are highly encouraged. Position papers on drug treatment of neurological or psychiatric disorders or other key topics in clinical neuroscience will also be considered for publication. I hope that in 2024, Current Neuropharmacology will continue to thrive and further enhance its quality and prestige.

*Current Neuropharmacology* has published the following Special Issues in 2023:

• **Understanding Brain Diseases: From Receptor Dysregulation to Neurodegeneration, Neuroinflammation and Memory Impairment**

Fabiola M. Ribeiro

• **Emerging Neurobiological Concepts and Therapies for Neurological Rare Diseases**

Panagiotis Bargiotas

• **Recent Medicinal Chemistry Studies against Neurodegenerative Diseases**

*Luciana Scotti* and *Marcus T. Scotti*

• **Mitochondrial Medicine for Neurological Disorders**

*Md. Sahab Uddin, Badrah S. Alghamdi* and *Ghulam Md. Ashraf*

• **Pediatric Bipolar Disorder: Evolution in Clinical and Biological Markers and Future Perspectives**

*Kirti Saxena, Kiki Chang* and *Gabriele Sani*

• **Epilepsy and Related Neuropsychiatric Comorbidities: Basic and Clinical Research**

*Rita Citraro* and *Antonio Leo*

• **From Phenomes to Genes: Phenotype-based Strategies in Rodents for Research on the Neurobiological and Genetic Bases of Psychiatric-relevant Traits**

Alberto Fernández-Teruel

• **Rethinking ‘Innovation’ in Psychiatry with Older and Newer Treatments: From Bench to Benchside**

*Domenico De Berardis* and *Giovanni Martinotti*

Upcoming Thematic Issues in 2024:

• **“Stress & Stress-related Disorders”**

Agorastos Agorastos

• **Is Pain a Symptom or a Disease? How Does the New Evidence Help to Better Understand this Unsolved Question?**

Massimo Allegri

• **The Subcortical Contribution to the Role of the Basal Ganglia in Action Selection**

*Véronique Coizet* and *Frederic Ambroggi*

• **Multi-targets of the Neurodegenerative Diseases**

Luciana Scotti

• **Advanced Progress in Neuroimaging Markers for Neurological and Psychiatric Disorders**

Peipeng Liang

• **Neurotrauma-induced Alzheimer’s Disease: A Closer Look**

Brandon Peter Lucke-Wold

• **Addiction: A Multi-faceted Issue**

Fabrizio Schifano

I wish you a Happy New Year!

